# Rinderpest in the Philippines

**Published:** 1901-05

**Authors:** 


### RINDERPEST IN THE PHILIPPINES.

On account of the reported existence of rinderpest in the Philippine Islands, Secretary Wilson some time ago recommended to the Secretary of War that such action be taken as might be necessary to prevent the introduction of the contagion into the Hawaiian Islands and the United States by animals brought on government transports. He also requested the Secretary of the Treasury to direct that special precautions be taken on the Pacific Coast to prevent the landing of susceptible animals without their being turned over to the Department of Agriculture for quarantine under the supervision of the Bureau of Animal Industry. Secretary Wilson's application was favorably considered.

Rinderpest is the great plague of Oriental countries, which has frequently swept over Europe, destroying nearly all bovine animals, and has attracted special attention during the past two or three years by its ravages in Africa, where in many sections it destroyed from 90 to 95 per cent. of all the cattle. Although inoculation has so far been efficacious as to somewhat reduce the losses from this disease, it still remains one of the most fatal to cattle.

